# Validation of Asian Body Mass Index Cutoff Values for the Classification of Malnutrition Severity According to the Global Leadership Initiative on Malnutrition Criteria in Patients with Chronic Obstructive Pulmonary Disease Exacerbations

**DOI:** 10.3390/nu14224746

**Published:** 2022-11-10

**Authors:** Yuka Shirai, Ryo Momosaki, Yoji Kokura, Yuki Kato, Yoshinaga Okugawa, Akio Shimizu

**Affiliations:** 1 Department of Rehabilitation Medicine, Mie University Graduate School of Medicine, Tsu 514-8507, Mie, Japan; 2 Clinical Nutrition Unit, Hamamatsu University Hospital, 1-20-1 Handayama, Higashi-ku, Hamamatsu 431-3192, Shizuoka, Japan; 3 Department of Nutritional Management, Keiju Hatogaoka Integrated Facility for Medical and Long-Term Care, Hosu 927-0023, Ishikawa, Japan; 4 Department of Genomic Medicine, Mie University Hospital, Tsu 514-8507, Mie, Japan; 5 Department of Gastrointestinal and Pediatric Surgery, Division of Reparative Medicine, Institute of Life Sciences, Mie University Graduate School of Medicine, Tsu 514-8507, Mie, Japan; 6 Department of Health Science, Faculty of Health and Human Development, The University of Nagano, 8-49-7, Nagano 380-8525, Nagano, Japan

**Keywords:** GLIM criteria, malnutrition, BMI, acute COPD exacerbation

## Abstract

Low body mass index (BMI) is an independent predictor of prolonged hospital stay and mortality in patients with chronic obstructive pulmonary disease (COPD). However, to the best of our knowledge, no studies have examined the validity of Asian BMI cutoff values for classifying severity based on the Global Leadership Initiative on Malnutrition (GLIM) criteria in patients with acute COPD exacerbations. This study sought to validate whether Asian BMI cutoff values can accurately predict 30-day in-hospital mortality, length of stay, and 90-day readmission outcomes for patients with acute COPD exacerbations. The present retrospective cohort study was conducted using a large claims database created by the JMDC. Patients were classified into three groups according to the severity of low BMI assessed using Asian BMI cutoff values. As a result, 624 (29.4%) had severely low BMI, and 444 (20.9%) had moderately low BMI. The severity of low BMI, as assessed by the Asian BMI cutoff values used in the GLIM criteria, was independently associated with 30-day in-hospital mortality (moderately low BMI: HR, 1.87; 95% CI, 1.13–3.08; *p* = 0.014 and severely low BMI: HR, 2.55; 95% CI, 1.66–3.92; *p* < 0.001). The Asian BMI cutoff values used to classify the severity of malnutrition in the GLIM criteria are clinically functional for predicting the prognosis of patients with acute COPD exacerbations.

## 1. Introduction

Around 12% of the global population suffers from chronic obstructive pulmonary disease (COPD), which has high morbidity and mortality rates and is a significant medical burden [1,2]. In line with this, acute COPD exacerbation requiring additional treatment for respiratory symptoms has been identified as a factor for poor prognosis [3]. Reports have shown that 22–40% of patients with COPD experience acute exacerbations at least once a year, with an associated in-hospital mortality rate of 15% or higher [3]. Therefore, better outcomes, such as fewer readmissions and deaths, are promoted by the early detection of patients who are at high risk for acute COPD exacerbations.

Studies have shown that patients with COPD frequently experience changes in body composition [4,5,6], with 30%–60% of affected patients presenting with malnutrition [7,8,9]. Malnutrition is a reliable predictor of extended hospital stays and 1-year mortality in patients with COPD [10,11]. Based on these findings, it is recommended that nutritional management be included in treatment programs for patients with COPD [12]. It is crucial to assess the nutritional status with appropriate screening and assessment tools and to identify patients who are malnourished to perform nutritional management that enhances therapeutic efficacy [13]. The National Institute for Health and Care Excellence (NICE) guidelines recommend that patients with COPD routinely calculate their BMI and seek advice from a registered dietitian if their BMI is abnormal (high or low) or changes over time [14]. A review of the role of nutritional therapy in treating COPD also indicated that nutritional screening assesses weight loss in addition to BMI [15]. Based on these findings, the definition of malnutrition for patients with COPD varies in the literature and remains controversial. It is unclear which nutritional screening or assessment tools are appropriate for patients with COPD. The use of various nutritional assessment criteria contributes to variations in the prevalence of malnutrition and may be a negative factor for COPD re-exacerbation and treatment. Therefore, we believe that identifying effective nutritional assessment tools for each disease state and the patient background and conducting consistent assessments will lead to effective nutritional interventions. In 2018, the Global Leadership Initiative on Malnutrition (GLIM) criteria were published as nutritional diagnostic criteria that considered the background of subjects, such as age and race [16]. An increasing number of reports have examined the effectiveness of these GLIM criteria and proved their effectiveness in predicting long- and short-term prognoses [17,18]. The severity classification in the GLIM criteria is determined using phenotypic criteria. One of the phenotypic criteria includes BMI, which can be based on race and age. Several studies have looked into the relationship between the GLIM criteria’s severity classification and prognosis utilizing cutoff BMI values for Asians [19]. A report on patients admitted to a university hospital in Japan and pneumonia showed an association between Asian BMI cutoff values for classifying severity in GLIM criteria with mortality and prolonged hospital stay [19,20]. Additionally, a European investigation on COPD patients revealed that malnutrition that met the GLIM criteria was linked to a higher likelihood of hospitalization and a longer length of stay [11,21]. However, no studies have used Asian threshold BMI values to categorize the degree of malnutrition in patients with acute COPD exacerbations using the GLIM criteria. Consequently, it is still unknown if the Asian BMI cutoff values, which have been used to categorize malnutrition severity in accordance with the GLIM criteria, are adequate for patients with acute COPD exacerbations.

The purpose of this study was to determine whether it was possible to accurately predict 30-day in-hospital mortality, length of stay, and 90-day readmission outcomes in patients with acute COPD exacerbations by using Asian cutoff BMI values for the classification of malnutrition severity according to the GLIM criteria.

## 2. Materials and Methods

### 2.1. Data Source

The present retrospective cohort study was conducted using a large claims database of insured persons’ ledgers, insurance claims, and health examination results collected from Japanese health insurance societies created by the JMDC since January 2005. The number of insured persons in this database was approximately 9.8 million as of the end of June 2020 [22]. This database also includes the Diagnostic Procedure Combination database of acute care hospitals in Japan. We extracted data from this database for patients hospitalized with COPD between April 2014 and August 2020. Primary diagnostic names and comorbidities were recorded using the International Statistical Classification of Diseases and Related Health Problems, 10th Revision (ICD-10) code. The database also includes demographic information, such as age, sex, height, and body weight. Given that the database is open to the public, Mie University’s ethics committee decided that an ethics review was unnecessary. Informed consent was waived due to the anonymization of the data provided by the JMDC.

The following data were extracted from the database. Comorbidities were assessed using the Charlson Comorbidity Index (CCI). The CCI is rated on a scale of 1–6 for 19 chronic diseases, including diabetes, congestive heart failure, and kidney disease, with higher scores for death-related diseases [23]. The Hugh–Jones dyspnea scale was used to assess physical symptoms associated with respiratory failure. This scale classifies patients into five stages based on motor function and dyspnea [24]. The Japan coma scale (JCS), indicated by Alert (0), Dull (1-digit code: 1, 2, 3), Somnolence (2-digit code: 10, 20, 30), and Coma (3-digit code: 100, 200, 300), is a widely used tool for assessing consciousness levels in Japan. The Barthel Index (BI) was used to assess patients’ Activities of Daily Living (ADL), with higher scores indicating greater independence in ADL [25]. The smoking index is the number of cigarettes smoked per day multiplied by the number of years of smoking, with a smoking index of 400 or more indicating increased risk of developing COPD [26,27].

### 2.2. Patient Selection

We enrolled patients hospitalized with COPD (ICD-10 code: J41–J44) from 1 April 2014 to 31 August 2020. Acute COPD exacerbation can be accompanied by acute, severe chest pain and severe dyspnea, which is a highly emergent status. Therefore, we hypothesized that cases transported by ambulance with COPD that led to hospitalization were acute COPD exacerbations. The Hugh–Jones dyspnea scale score was used to determine COPD severity, and patients with missing BMI data were excluded.

### 2.3. BMI Cutoff Values for Asians to Classify the Severity of GLIM-Defined Malnutrition

The BMI cutoff values for Asians proposed by Maeda et al. were adopted for classifying the malnutrition severity as defined by the GLIM criteria [19]. In previous studies, these BMI cutoff values were validated in university hospital inpatients [19] and in elderly patients with pneumonia [20] and were associated with increased mortality, longer hospital stays, and increased readmission rates. The BMI cutoff values for Asians for classifying the GLIM-defined malnutrition severity were as follows:

Moderately low BMI: BMI 17.0–18.4 if aged below 70 years and BMI 17.8–19.9 if aged above.

Severely low BMI: BMI < 17.0 if aged below 70 years and BMI < 17.8 if aged above 70 years.

These BMI cutoff values were used to classify low BMI into two grades (moderately low BMI and severely low). Furthermore, patients who did not meet the above Asian BMI cutoff values were included in the control group.

### 2.4. Outcomes

The primary outcome was 30-day in-hospital mortality, whereas the secondary outcomes were length of stay and readmission rate within 90 days due to the acute exacerbation of COPD. Approximately 70% of the patients with acute COPD exacerbations who were readmitted did so within 90 days [28]. Therefore, readmission was defined as hospitalization within 90 days of discharge. In addition, patients were readmitted to the same hospital they were originally admitted.

### 2.5. Statistical Analyses

Baseline data and outcomes were compared between the no-malnutrition, moderate malnutrition, and severe malnutrition groups. Categorical variables are presented as the number of subjects (%) and median (interquartile range), and comparisons between groups were performed using the chi-square test. Continuous variables are presented as mean ± SD, and analysis of variance was used for comparison between groups. Kaplan–Meier curves were constructed to clarify the association between BMI-based nutritional status and 30-day in-hospital mortality. Differences in survival rates based on nutritional status were evaluated using log-rank analysis. Cox proportional hazards regression models were used to assess associations between 30-day in-hospital mortality and variables. Moreover, the association between variables and length of stay and readmission within 90 days was evaluated using a linear regression model and binomial logistic regression analysis. The following variables were included in the model based on previous studies on patients with pulmonary disease: age (category: 64 years or younger, 65–74 years, 75–89 years, and 90 years or older), sex, comorbidities, JCS, smoking index, respiratory use on admission, Hugh–Jones dyspnea scale score, BI, number of beds, and year of admission. All statistical analyses were performed using the SPSS version 21.0 (IBM, Tokyo, Japan). Statistical significance was defined as *p* < 0.05.

## 3. Results

**Figure 1:**
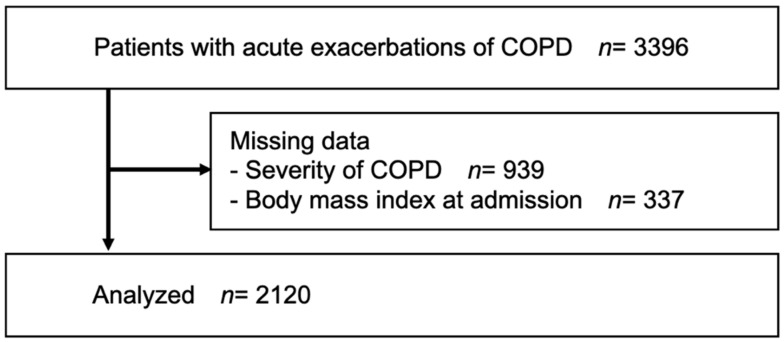
Flowchart of the sample size in this study.

The study examined data from 3396 patients with acute COPD exacerbations. A total of 1276 patients were excluded due to missing data (Hugh–Jones dyspnea scale score: 939; BMI at admission: 337). Thus, 2120 persons were ultimately analyzed (mean age 77.4 ± 9.3 years; 19.5% female) (Figure 1).

### 3.1. Baseline Characteristics

The baseline characteristics of the patients are presented in Table 1. Accordingly, 624 (29.4%) and 444 (20.9%) had severely and moderately low BMI according to the Asian cutoff for BMI in the GLIM criteria. The mean BMI by severity was 15.68 ± 1.41 kg/m^2^ for severely low BMI and 18.68 ± 0.73 kg/m^2^ for moderately low BMI. Regarding the JCS, severely low BMI had a higher rate of 100–300 (coma) than other groups, and more patients had a low level of consciousness (*n* = 19, rate within group 3.0%, severely low BMI vs. *n* = 6, rate within group 1.4%, moderate low BMI vs. *n* = 18, rate within group 1.7%, control; *p* = 0.018).

### 3.2. A Comparison of Outcomes

Table 2 compares outcomes by the severity of low BMI in Asians. The severely low BMI group had a higher 30-day in-hospital mortality than the moderately low BMI and control groups (*n* = 62, rate within group 9.9%, *p* < 0.001). The length of stay extended as the severity of malnutrition progresses (15.0 [9.0–26.0], severely low BMI vs. 13.0 [8.0–23.0], moderately low BMI vs. 12.0 [7.0–20.0], control; *p* < 0.001). The 90-day readmission rate was the highest in the moderately low BMI group (*n* = 18, rate within group 3.3%, severely low BMI vs. *n* = 15, rate within group 3.8%, moderately low BMI vs. *n* = 11, rate within group 1.1%, control; *p* = 0.002).

### 3.3. 30-Day In-Hospital Mortality

**Figure 2:**
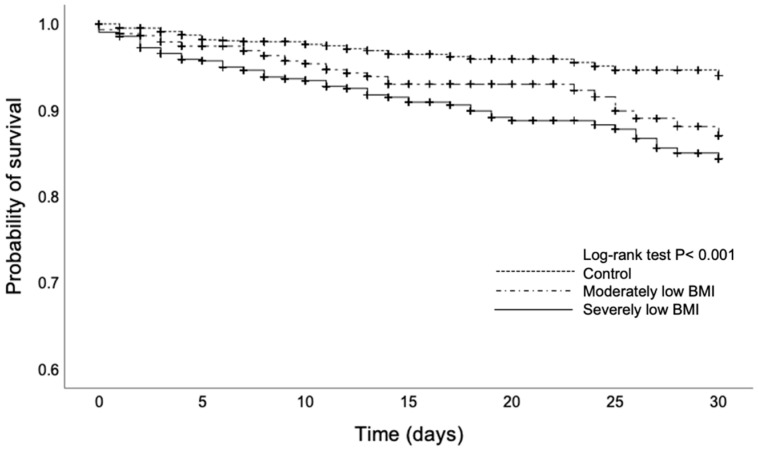
Survival curves for 30-day in-hospital mortality.

The Kaplan–Meier curves for the 30-day in-hospital mortality according to severity based on Asian BMI cutoff values are shown in Figure 2. The severely low BMI group had a significantly higher 30-day in-hospital mortality than the moderately low BMI and control groups (log-rank test, *p* < 0.001).

Table 3 shows the results of Cox proportional hazards analyses of 30-day in-hospital mortality. Severity based on BMI cutoff values among Asians used in the study was independently associated with 30-day in-hospital mortality (moderately low BMI: HR, 1.87; 95% CI, 1.13–3.08; *p* = 0.014 and severely low BMI: HR, 2.55; 95% CI, 1.66–3.92; *p* < 0.001).

### 3.4. Length of Stay and 90-Day Readmission Rate

Table 4 and Table 5 show the results of the multivariate analysis on length of stay and 90-day readmission rate. Severely low BMI was independently associated with a longer length of stay (coefficient, 3.40; 95% CI, 1.51–5.29; *p* < 0.001). However, moderately low BMI was not significantly associated with a longer length of stay (coefficient, 1.57; 95% CI, −0.52–3.67; *p* = 0.141). The severity of low BMI according to Asian cutoff values proposed in the GLIM criteria was independently associated with 90-day readmission (moderately low BMI: odds ratio, 3.68: 95% CI, 1.63–8.31; *p* = 0.002; severely low BMI: odds ratio, 2.75; 95% CI, 1.26–6.02; *p* = 0.011, respectively).

## 4. Discussion

This study examined the validity of Asian cutoff BMI values for classifying malnutrition severity according to the GLIM criteria for patients with acute COPD exacerbation using data from all over Japan. According to our findings, 30-day in-hospital mortality was independently correlated with Asian BMI cutoff values, which categorize malnutrition severity using the GLIM criteria. The longer length of hospital stay was significantly associated only with severely low BMI. Additionally, the severity classified according to these BMI cutoffs was independently associated with readmission within 90 days.

This study showed that the 30-day in-hospital mortality was independently correlated with Asian BMI cutoff values. This outcome is comparable to that described in earlier investigations, which demonstrated an independent relationship between low BMI and mortality in patients with COPD [29,30]. Compared to the BMI 20.0–22.0 kg/m^2^ group, the BMI < 18.5 kg/m^2^ group had increased mortality rates in research on Japanese male patients with COPD [30]. Similarly, among Korean patients with COPD, BMI < 18.5 kg/m^2^ was associated with the highest mortality [31]. In these studies, the BMI < 18.5 kg/m^2^ group had a mean BMI range of 17.0–17.5 kg/m^2^, which is close to the severely low BMI assessed by the Asian cutoff used in this study. Moreover, research on patients with pneumonia that employed a BMI cutoff comparable to that used here found that the severity of malnutrition was independently related to 30-day in-hospital mortality [20]. These findings suggest that a BMI < 18.5 kg/m^2^ may increase mortality in patients with respiratory diseases. Hence, adapting the Asian BMI cutoffs for GLIM criteria severity classification to patients with COPD exacerbations may more sensitively predict patients at a high risk of death.

In patients with acute COPD exacerbations, the cutoff value indicating severely low BMI in Asians was independently linked to a longer length of stay. Severely low BMI may have decreased ADL, promoting a prolonged hospital stay. This study showed that ADL on admission tended to be lower with the increase in severity based on Asian BMI cutoff values. In fact, a study on Japanese patients with COPD reported that as BMI decreases, the degree of ADL independence decreases and the length of hospital stay increases [32]. Moreover, a study on pneumonia patients using the same BMI cutoff as in the present study revealed that the severity of malnutrition was independently correlated with an extended length of hospital stay [20]. In addition, similar to this study, ADL on admission was significantly lower in patients with severe malnutrition, arguing that a decrease in ADL may have prolonged hospital stay. These findings revealed that a severely low BMI caused a decrease in ADL, which extended the length of stay. Among patients with acute COPD exacerbations, a severely low BMI using the Asian BMI cutoff values to classify the severity based on the GLIM criteria may predict a prolonged length of stay.

The current study also demonstrated that 90-day readmission rates in patients with acute COPD exacerbations were independently correlated with Asian BMI cutoff values. This outcome was consistent with earlier studies that demonstrated a link between decreased BMI and higher readmission rates in COPD patients [32,33]. Patients assessed as having low BMI according to the BMI cutoff used in this study were more likely to develop COPD exacerbations after discharge, which may have led to readmission. Reports have shown that a BMI of <20 kg/m^2^ reduces the time to COPD exacerbation [12], suggesting that a low BMI can be a risk factor for COPD exacerbation. In this study, the cutoff value indicating severely low BMI was <20 kg/m^2^. Therefore, in patients with acute COPD exacerbations, the Asian BMI cutoff values used for severity classification based on the GLIM criteria were shown to result in COPD exacerbations after discharge and were associated with readmission. Furthermore, our multivariate analysis found that moderately low BMI was associated with a higher risk of readmission compared to severely low BMI. In a report examining the effects of longitudinal changes in BMI in Korean patients with COPD, a reduction in BMI was independently associated with COPD exacerbation [31]. Patients with COPD have been considered more likely to have skeletal muscle and adipose tissue loss over time, which can promote poor clinical outcomes and increased readmission. In the mentioned study, the group with a reduction in BMI included patients with a baseline BMI of 18.5–24.9 or 25.0–29.9 kg/m^2^, with no patients having a BMI < 18.5 kg/m^2^. The mean BMI of the patients with moderately and severely low BMI in the current study was 18.68 and 15.68 kg/m^2^, respectively. Those with moderately low BMI with a higher baseline BMI than those with severely low BMI may have been more likely to experience a reduction in BMI. These findings imply that moderately low BMI may have worsened clinical outcomes because of the BMI loss, which raised the likelihood of readmission.

This study shows a correlation between outcomes, such as length of stay and mortality in patients with acute COPD exacerbations, and Asian BMI cutoff values for determining the degree of malnutrition. The BMI cutoff values for Asians are indicators of low BMI and are used to classify nutritional status by severity. It has been suggested that molecular mechanistic changes lead to a decrease in skeletal muscle mass in patients with COPD with a BMI < 18.5 kg/m^2^ [34]. However, a previous study of overweight (BMI 25–29.9 kg/m^2^) and obese (BMI > 30 kg/m^2^) patients with COPD reported that about half of them had muscle mass below the cutoff values [35]. The loss of skeletal muscle mass causes a deterioration in physical function and an increase in the inflammatory state, thus slowing down the healing process [36,37]. In this study, the control group had a BMI of 23.12 ± 3.01 kg/m^2^, so only a limited number of patients were eligible for overweight and obesity. Therefore, the findings of this study may have drawn attention to the link between low BMI below the Asian BMI cutoff and longer hospital admissions and mortality.

This study’s strength is that it is founded on a sizable sample from an extensive national database. We believe that this is the first study to validate Asian BMI cutoff values for GLIM criteria severity classification among patients with acute COPD exacerbations. Nonetheless, there are a few limitations to this study that should be mentioned. The Hugh–Jones dyspnea scale used in this study assesses physical symptoms associated with respiratory failure and may not always correspond to the acute COPD exacerbation severity. Second, it should be noted that this study examines the validity of Asian BMI cutoff values for severity classification under the GLIM criteria for patients with acute COPD exacerbations and does not validate the GLIM criteria. Additionally, few reports have examined the efficacy of the GLIM criteria in Asian patients with acute COPD exacerbations. In the future, it is necessary to investigate whether GLIM criteria using BMI cutoff values in Asians are effective in predicting prognosis.

## 5. Conclusions

The current study showed that Asian cutoff values for BMI, which is used to classify malnutrition severity in the GLIM criteria, was independently associated with 30-day in-hospital mortality and 90-day readmission among patients with acute COPD exacerbations. In addition, prolonged length of stay was independently associated with severely low BMI. In clinical practice, an appropriate, noninvasive, and convenient cutoff for BMI is critical. The Asian BMI cutoff value used for GLIM criteria severity classification is clinically useful for predicting the prognosis of patients with acute COPD exacerbations.

## Figures and Tables

**Table 1:** Baseline characteristics of patients with acute exacerbations of COPD by the severity of low BMI in Asians.

Variables	Severely Low BMI (*n* = 624)	Moderately Low BMI (*n* = 444)	Control (*n* = 1052)	*p*-Value
Female sex, *n* (%)	147 (23.6)	75 (16.9)	192 (18.3)	0.009
Age, years				<0.001
≤64	33 (5.3)	20 (4.5)	106 (10.1)	
65–74	177 (28.4)	101 (22.7)	288 (27.4)	
75–89	358 (57.4)	278 (62.6)	549 (52.2)	
≥90	56 (9.0)	45 (10.1)	109 (10.4)	
Body mass index, kg/m^2^	15.68 ± 1.41	18.68 ± 0.73	23.12 ± 3.01	<0.001
Charlson Comorbidity Index, points	1.0 [1.0–2.0]	1.0 [1.0–2.0]	2.0 [1.0–2.0]	0.367
Japan coma scale at admission, *n* (%)				0.018
0 (Alert)	489 (78.4)	351 (79.1)	855 (81.3)	
1–3 (Dull)	82 (13.1)	69 (15.5)	151 (14.4)	
10–30 (Somnolence)	27 (4.3)	10 (2.3)	19 (1.8)	
100–300 (Coma)	19 (3.0)	6 (1.4)	18 (1.7)	
Missing	7 (1.1)	8 (1.8)	9 (0.9)	
Smoking index				0.757
None	156 (25.0)	108 (24.3)	278 (26.4)	
1–499	78 (12.5)	50 (11.3)	120 (11.4)	
500–999	142 (22.8)	100 (22.5)	255 (24.2)	
≥1000	171 (27.4)	138 (31.1)	275 (26.1)	
Unspecified/missing	77 (12.3)	48 (10.8)	124 (11.8)	
Use of ventilator at admission, *n* (%)	116 (18.6)	63 (14.2)	130 (12.4)	0.002
Hugh–Jones dyspnea scale, *n* (%)				<0.001
1	38 (6.1)	32 (7.2)	91 (8.7)	
2	51 (8.2)	41 (9.2)	112 (10.6)	
3	58 (9.3)	35 (7.9)	150 (14.3)	
4	161 (25.8)	107 (24.1)	261 (24.8)	
5	316 (50.6)	229 (51.6)	438 (41.6)	
Barthel Index at admission, *n* (%)				0.093
0–20	173 (27.7)	113 (25.5)	242 (23.0)	
25–45	89 (14.3)	56 (12.6)	111 (10.6)	
50–70	109 (17.5)	77 (17.3)	185 (17.6)	
75–95	45 (7.2)	42 (9.5)	104 (9.9)	
100	112 (17.9)	81 (18.2)	232 (22.1)	
Missing	96 (15.4)	75 (16.9)	178 (16.9)	
Number of beds, *n* (%)				0.187
20–99	13 (2.1)	10 (2.3)	15 (1.4)	
100–199	156 (25.0)	111 (25.0)	213 (20.2)	
200–299	86 (13.8)	59 (13.3)	158 (15.0)	
300–499	245 (39.3)	174 (39.2)	415 (39.4)	
≥500	124 (19.9)	90 (20.3)	251 (23.9)	
Year of admission, *n* (%)				0.935
2014	30 (4.8)	25 (5.6)	41 (3.9)	
2015	60 (9.6)	39 (8.8)	95 (9.0)	
2016	79 (12.7)	54 (12.2)	150 (14.3)	
2017	95 (15.2)	68 (15.3)	175 (16.6)	
2018	143 (22.9)	106 (23.9)	247 (23.5)	
2019	156 (25.0)	105 (23.6)	248 (23.6)	
2020	61 (9.8)	47 (10.6)	96 (9.1)	

Abbreviation: COPD, chronic obstructive pulmonary disease.

**Table 2:** Comparison of outcomes by the severity of low BMI in Asians.

Variables	Severely Low BMI	Moderately low BMI	Control	*p*-Value
30-day in-hospital mortality, *n* (%)	62 (9.9)	31 (7.0)	34 (3.2)	<0.001
Length of hospital stay in survival cases, d	15.0 [9.0–26.0]	13.0 [8.0–23.0]	12.0 [7.0–20.0]	<0.001
90-day readmission in survival cases, *n* (%)	18 (3.3)	15 (3.8)	11 (1.1)	0.002

**Table 3:** Cox proportional hazards analyses for 30-day in-hospital mortality.

Variables	Hazard Ratio	95% Confidence Interval	*p*-Value
Nutrition status			
Control (reference)	–	–	–
Moderately low BMI	1.87	1.13–3.08	0.014
Severely low BMI	2.55	1.66–3.92	<0.001

Models were adjusted for the following variables: age, sex, Charlson Comorbidity Index, JCS at admission, smoking history, use of the ventilator at admission, Hugh–Jones dyspnea scale, Barthel Index at admission, number of beds, and year of admission.

**Table 4:** Linear regression analyses for the length of hospital stay in survival cases.

Variables	Coefficient	95% Confidence Interval	*p*-Value
Nutrition status			
Control (reference)	–	–	–
Moderately low BMI	1.57	−0.52–3.67	0.141
Severely low BMI	3.40	1.51–5.29	<0.001

Models were adjusted for the following variables: age, sex, Charlson Comorbidity Index, JCS at admission, smoking history, use of the ventilator at admission, Hugh–Jones dyspnea scale, Barthel Index at admission, number of beds, and year of admission.

**Table 5:** Logistic regression analysis for 90-day readmission in survival cases.

Variables	Odds Ratio	95% Confidence Interval	*p*-Value
Nutrition status			
Control (reference)	–	–	–
Moderately low BMI	3.68	1.63–8.31	0.002
Severely low BMI	2.75	1.26–6.02	0.011

Models were adjusted for the following variables: age, sex, Charlson Comorbidity Index, Japan coma scale at admission, smoking history, use of the ventilator at admission, Hugh–Jones dyspnea scale, Barthel Index at admission, number of beds, and year of admission.

## Data Availability

The datasets generated and/or analyzed during the current study are available from the corresponding on reasonable request.
